# Post-calving umbilical cord tissue offcut: A potential source for the isolation of bovine mesenchymal stem cells

**DOI:** 10.14202/vetworld.2020.2772-2779

**Published:** 2020-12-25

**Authors:** Parishma Debbarma, Tanmay Mondal, Camelia Manna, Kuldeep Kumar, Joydip Mukherjee, Bikash Chandra Das, Sadhan Bag, Kinsuk Das

**Affiliations:** 1 Department of Veterinary Physiology, West Bengal University of Animal and Fishery Sciences, Kolkata, West Bengal, India; 2 Division of Physiology and Climatology, ICAR-Indian Veterinary Research Institute, Izatnagar, Uttar Pradesh, India; 3 Eastern Regional Station, ICAR-Indian Veterinary Research Institute, Kolkata, West Bengal, India

**Keywords:** cattle, umbilical cord tissue, mesenchymal stem cells, isolation

## Abstract

**Background and Aim:**

Veterinary health care is an emergent area in animal sciences and innovative therapeutic approaches happen to be imperative in the present days. In view of the importance of cattle health and production, it is necessary to take up contemporary approach of stem cell therapy in this sector also. This study aimed to standardize an explant culture method of bovine umbilical tissue offcut to isolate mesenchymal stem cells (MSCs) because considerable efforts are required for ensuring easy accessibility and availability of MSCs in bulk quantity, as well as in establishing and characterizing the cell lines.

**Materials and Methods:**

The umbilical cord (UC) tissue matrix offcut was collected after calving. A simplified *in vitro* cell isolation technique was followed to collect the emerged out cells from the explants of UC. Further, we expanded these isolated cells *in vitro*, observed its growth kinetics, and characterized to confirm as per the criterion of bovine MSCs.

**Results:**

A considerable exponential growth rate of the UC-derived cells was noticed. In addition to their confirmation as MSCs, the cells also exhibited plastic adherent property and maintained the spindle-shaped morphology throughout the *in vitro* culture. The cultured cells were found positive MSC-specific surface markers CD105, CD90, and CD73 and were negative for hematopoietic cell marker CD45. Cytochemical studies revealed the ability of the cells to differentiate into osteogenic, chondrogenic, and adipogenic lineages.

**Conclusion:**

This simplified method of isolation and culture of bovine multipotent MSCs from the UC offcut collected after calving could be extrapolated for the greater availability of the cells for prospective therapeutic applications.

## Introduction

Stem cells are the specific group of primitive cells which maintain the capacity to renew themselves by cell division and have the ability to differentiate into different adult cells. Mesenchymal stem cells (MSCs) are adult stem cells of mesodermal origin. The decades of stem cell research have explored that MSCs are highly proliferative with an ability to differentiate into a variety of connective tissues in response to proper biological cues. Therefore, these cells are considered as one of the choices in stem cell-based tissue regeneration. MSCs can be isolated from a range of adult tissues. Moreover, the components of fetal adnexa such as amniotic fluid, amniotic membrane, and umbilical cord (UC) tissue matrix are also the potential sources of MSCs [1,2]. The MSCs isolated from cord tissues have the advantages of easy to harvest through non-invasive method without any ethical issue or risk to the donor, can be expanded *in vitro* and cryogenically stored, genetically modified and differentiated *in vitro* for therapeutic purpose. Till date, the MSCs have been successfully derived from caprine, bubaline, canine, equine, and porcine UC matrix [3-6].

The larger animal models are sometimes preferred over the traditional laboratory animals in various fields of experimental medicine.Domestic cattle (*Bos Taurus*) have similarities with human in terms of many of the anatomical and physiological features including a long life span. Therefore, the stem cells isolated from bovine species could be an alternative cellular model in regenerative medicine. Moreover, exploring the stem cells biology of domestic animals has its own characteristics, in particular has importance in veterinary regenerative therapy. A considerable achievement has been noticed in recent years about the isolation and characterization of MSCs from different domestic and pet animals with the perspective of therapeutic applications. In spite of ample relevance of the experimental bovine model in both human and veterinary medicines, the prospect of MSCs isolated from cattle has been least explored till now.

Bovine MSCs (bMSCs) have been isolated mostly from bone marrow and adipose tissue for various experiments till date [7-10]. Among the fetal origin, MSCs have been isolated from bovine amniotic fluid, UC blood, even recently from placenta [11-13]. Cardoso *et al*. [14] made an attempt to isolate bMSCs from Wharton’s jelly (WJ) and maintained in 3D serum-free condition which guided the scientific community toward utilizing cord tissue as a readily accessible potential source of bMSCs. Later on, Xiong *et al*. [15] and Silva *et al*. [16] showed the possibility of isolating these cells by *in vitro* culture system. However, to make the cells more accessible on large scale by minimizing ethical concern, it is necessary to standardize a non-invasive isolation technique of bMSCs.

This study aimed to narrate the procedure of isolation of bMSCs by the explant culture of the offcut UC tissue matrix followed by its propagation, expansion, and characterization *in vitro*. The projected method could be extrapolated for the larger accessibility of the bMSCs for prospective therapeutic applications in bovine medicine.

## Materials and Methods

### Ethical approval and Informed consent

This is basically an *in vitro* cell culture work. The UCs were collected after calving from the animals came to the veterinary hospital as outdoor patients. The UCs were removed from the calf immediately after birth and discarded. Those offcuts of the cords were used for the isolation of cells after informed consent of the owners, under the supervision of registered veterinarians and by following all the safety compliances of the Institutional Animal Ethics Committee (IAEC) of West Bengal University of Animal and Fishery Sciences which has the approval (763/GO/Re/SL/03/ CPCSEA) of the Committee for the Purpose of Control and Supervision of Experiments on Animals (CPCSEA) under the Ministry of Fisheries, Animal Husbandry and Dairying, Government of India. With the directive of Animal Welfare Board of India, CPCSEA is a statutory committee of the Govt. of India established under Chapter 4, Section 15(1) of the Prevention of Cruelty to Animals Act 1960. Informed consent was obtained from the owners.

### Study period and location

The present study was conducted at the Research and Information Center of the Department of Veterinary Physiology under West Bengal University of Animal and Fishery Sciences, Belgachia campus, Kolkata during the period of January 2019 to January 2020.

### Collection of bovine UC

UCs used for different experiments are commonly collected at the time of parturition [14,16]. Here, in this study, the UCs were obtained from the nearby veterinary hospitals with informed consent of the owners and by following all the safety compliances of the IAEC. The UCs were collected from the calves (primiparous Holstein-Friesian crossbreed of average 2 years age, n=3) that had been delivered by cesarean section due to dystocia condition after a gestational age of 36-38 weeks. The fetus, after taken out from uterus, was held up and the UC was ligated with artery forceps on both sides and incised under aseptic condition. Cord blood was drained out immediately and fresh UC was collected in sterile container (HiMedia PW1148, India) with chilled cell culture grade phosphate-buffered saline pH 7.4 (HiMedia TL1101, India) supplemented with antibiotics and antimycotic penicillin (100 U/mL), streptomycin (100 μg/mL), and amphotericin B (0.25 μg/mL) (HiMedia A002, India). Samples were transported to the laboratory within 1 h of collection.

### Explant culture of UCs tissue matrix

**Figure-1:**
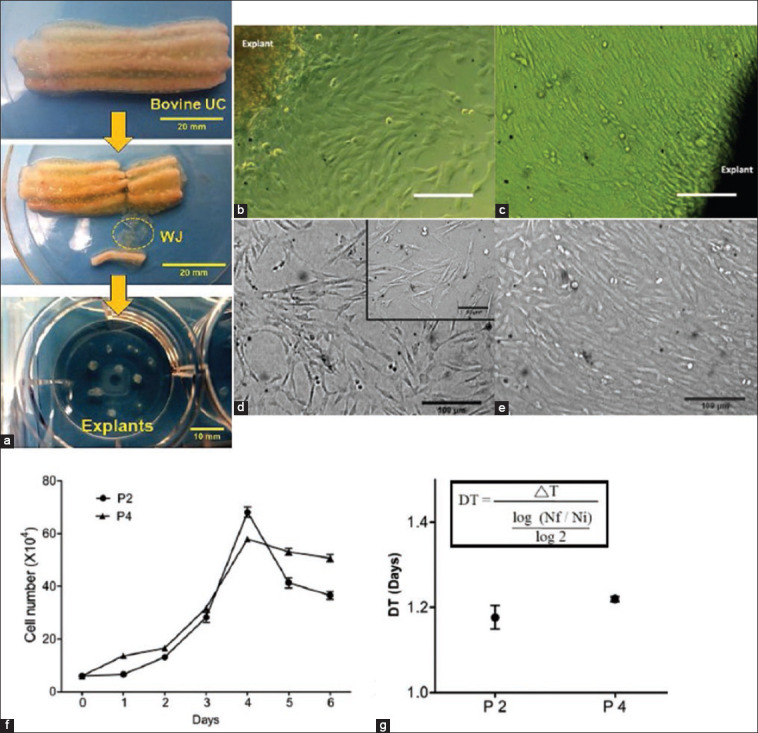
Isolation and expansion of bovine umbilical cord tissue-derived cells by Wharton’s jelly explant culture. (a) The flow chart illustrates the separation of Wharton’s jelly (WJ) from umbilical cord (UCs) followed by seeding of WJ explants in cell culture plate. (b) Phase contrast images of emerged out UCs derived cells from the edges of explants after 7 days and (c) after 10 days of culture in MSC medium. (d) Morphology of cells in P2 and (e) in P4 in confluence, scale bar (b, c, d, and e: 100 μm; inset: 50 μm). (f) Growth kinetics. (g) Cell doubling time studies in P2 and P4 cells.

The *in vitro* explant tissue culture is a reliable method for easy isolation of cells [14,16]. The UCs were carefully washed in sterile chilled phosphate-buffered saline (PBS) to rinse off the blood and blood clots within it, if any. Again to disinfect the cords were dipped into 70% ethanol for 30 s and washed thoroughly to remove any trace of ethanol residue. The UCs were then dissected to remove the blood vessels, and the underlying tissue matrix, that is, WJ was excised and minced into very fine pieces to make the explants of 3-5 mm each. Thereafter with the help of fine forceps, these explants were cautiously placed in tissue culture plates (HiMedia, TCP173, India) and allowed to air dry under a laminar hood for 5 min for attachment (Figure-1a). The explants were gently covered with MSC culture medium containing Dulbecco’s Modified Eagle Medium (DMEM)-low glucose media supplemented with 15% fetal bovine serum (FBS), L-glutamine (2 mM), penicillin (100 U/mL), streptomycin (100 μg/mL), and amphotericin B (0.25 μg/mL) (all are from HiMedia, AL183, RM9970, TCL012, A002, India), and were maintained at a humidified atmosphere of 37°C and 5% CO_2_ in an incubator (Eppendorf, Galaxy^®^ 48R, Germany). After 48 h, the medium was changed for the 1^st^ time with 2 ml of fresh warm MSC culture medium containing DMEM-low glucose media supplemented with 15% FBS, L-glutamine (2 mM), penicillin (100 U/mL), streptomycin (100 μg/mL), and amphotericin B (0.25 μg/mL) (all are from HiMedia AL183, RM9970, TCL012, A002; India). Thereafter, medium changes were given in every 48 h and the explants were maintained for another 10 days.

### Primary isolation and expansion of UCs derived cells

The explant culture of UCs for the isolation of MSCs has been performed for different *in vitro* cellular experiments [14,16]. Herein, the explant culture was maintained *in vitro* for around 10 days. Cultures were observed under phase-contrast microscope (Olympus, Japan) at a regular interval to check the emergence of cells from the explant edges and also its confluence. Once a sufficient number of cells emerged out from the explants and covered nearly 70% of the culture plate, the pieces were removed using blunt-end tips (HiMedia LA973, India). The attached cells were then trypsinized (0.25% trypsin-EDTA, HiMedia, TCL049, India) and replated at the density of 5000 cell/cm^2^, and maintained in MSC culture medium at 37°C in a humidified atmosphere of 5% CO_2_. For further expansion of the cell population, they were serially passaged by trypsinization after reaching 70-80% confluence. These cells were maintained up to passage 4 for further experiments.

### Growth kinetics and cell doubling time (DT) studies

These two experiments were done for both in passage 2 (P2) and passage 4 (P4) cells. Cells were seeded in 6-well plates at a density of 60,000 cells/well and maintained in MSCs culture medium for 6 days, with medium refreshment in every 48 h interval. At 24 h interval, after trypsinization followed by trypan blue dye exclusion test (Thermo Fisher, Cat.12250061, USA), the cell number was manually counted by hemocytometer in triplicate wells. A cell growth curve was prepared based on the calculation of the mean cell numbers.

Cell population DT was calculated from hemocytometer counts for passage 2 and 4 by the method as proposed by Vidal *et al*. [17] with the following formula: 
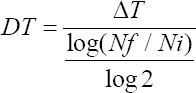
 where, ΔT is the cell culture time, Nf is the final number of cells, and Ni is the initial number of cells.

### Immunofluorescence staining with MSCs surface markers

MSC-specific positive surface markers and a negative marker were used for immunostaining [4,14]. The passage 4 cells were cultured *in vitro* onto the pre-cleaned and sterilized coverslips. After reaching the normal confluence, the cells were fixed in 4% paraformaldehyde at room temperature followed by PBS wash and then permeabilized in 0.25% Triton X-100. After thorough PBS washing, non-specific binding sites were blocked with 2% bovine serum albumin for 1 h. The cells were then incubated for overnight at 4°C with appropriate primary antibodies of MSC surface markers rabbit polyclonal anti-CD90 (1:100; Boster Biological, USA), rabbit polyclonal anti-CD105 (1:100; Boster Biological, USA), rabbit polyclonal anti-CD73 (1:100; Santa Cruz, USA), and rabbit polyclonal anti-CD45 (1:100; Santa Cruz, USA) as a negative marker. After brief PBS washing, they were further incubated with FITC-conjugated goat anti-rabbit secondary antibody (1:200; Santa Cruz, USA) for 4 h in darkness at room temperature. After washing the coverslips with PBS, they were mounted onto glass slide with DAPI ProLong^®^ Gold Antifade solution (Invitrogen, USA). Images were captured in inverted fluorescence microscope (Carl Zeiss, Germany) with AxioVision 4.0 image analysis system.

### Mesodermal lineage differentiation *in vitro*

The isolated cells were induced to differentiate toward osteogenic, chondrogenic, and adipogenic lineages by *in vitro* culture with specific differentiation medium for 21 days [14].

#### Osteogenic differentiation

For osteogenic differentiation, cells at passage 4 were cultured at plating density of 8×10^3^/cm^2^. The differentiation was induced by the HiOsteoXL™ Osteocyte Differentiation Medium (HiMedia, AL522, India). Cells were cultured for 21 days and medium was refreshed on every 3^rd^ day.

After 21 days of differentiation protocol, the culture plates were washed with PBS, fixed with 4% paraformaldehyde for 20 min at RT, and again washed with PBS to remove the fixative. Alizarin red cytochemical staining (Sigma; 0.2% solution, pH 4.1) was done at room temperature for 1 h in darkness. A gentle PBS wash was given to remove the excess stain. Accumulation of mineralized nodules of orange-red color was visualized by a phase-contrast microscope (Olympus, Japan).

#### Chondrogenic differentiation

For chondrogenic differentiation, cells at passage 4 were cultured at plating density of 8×10^3^/cm^2^. The differentiation was induced by the HiChondroXL™ Chondrocyte Differentiation Medium (HiMedia, AL523, India). Cells were cultured for 21 days and medium was refreshed on every 3^rd^ day.

Chondrogenic differentiation was confirmed by Alcian blue (Sigma, in 0.3% acetic acid, pH 2.5) cytochemical staining with the detection of proteoglycan stained in blue color and photographed by a phase-contrast microscope (Olympus, Japan).

#### Adipogenic differentiation

For adipogenic differentiation, cells at passage 4 were cultured at plating density of 8×10^3^/cm^2^. The differentiation was induced by the HiAdipoXL™ Adipocyte Differentiation Medium (HiMedia, AL521, India). Cells were cultured for 21 days and medium was refreshed on every 3^rd^ day.

Differentiation was evaluated after visualization of accumulated lipid droplets after the Oil red- O staining (Sigma, 0.1% in 60% isopropanol) and photographed by a phase-contrast microscope (Olympus, Japan).

## Results

### Explant culture of bovine UCs tissue matrix

The WJ isolated from bovine UCs tissue matrix was cultured in polystyrene-coated tissue culture plates under MSCs medium (Figure-1a). Spindle-shaped plastic adherent small-sized cells emerged out from the edges of explants after 7 days were further became confluent after 10 days and maintained their fibroblast-like morphology (Figure-1b and c). When the confluence reached, about 70-80% plates were trypsinized and replated in MSCs culture medium.

### Isolation and propagation of UCs derived cells

Cells isolated from explants were maintained in MSCs medium and expanded up to 4^th^ passage (P4). In initial passages (P2), the cells proliferated quickly in the form of small spindle-shaped fibroblasts (Figure-1d). The features of the cells were changed gradually to create a homogenous monolayer on culture surface in late passages P4 (Figure-1e). Therefore, in consecutive passages, the cells could able to maintain spindle-shaped fibroblastic morphology and adhered to the culture substrate.

### Growth kinetics study

This experiment was done by counting the adherent cells in the culture for a period of 6 days both in P2 and P4 cells and by plotting the data graphically as cell numbers versus time. The lag phase of growth was noticed in both the P2 and P4 cells up to first 24 h of seeding and thereafter an exponential phase up to 4^th^ day of culture. In case of P4, a stationary phase was noticed between day 4 and 6, whereas P2 population declined immediately after exponential phase (Figure-1f). In addition, the population DT up to the end of exponential phase in P2 and P4 cells was calculated as 1.1767±0.03 and 1.220±0.01 days, respectively (Figure-1g).

### Immunophenotypic expression of MSC markers in UCs derived cells

**Figure-2:**
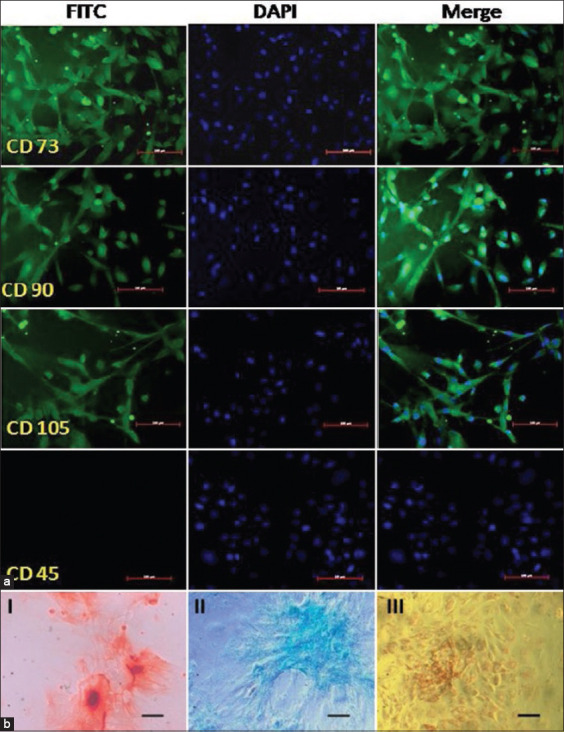
Characterization of bovine UCs derived MSCs. (a) Immunofluorescence staining of bovine UCs derived cells with MSC-specific surface markers (positive for CD105, CD90, and CD73 and negative for CD45); scale bar: 100 µm. (b) Cytochemical staining after 21 days of *in vitro* differentiation. (I) Osteocytes (Alizarin red staining), (II) chondrocytes (Alcian blue staining), and (III) adipocytes (Oil red- O staining); scale bar: 100 µm.

To confirm whether these UCs derived cells express the surface markers of MSCs, the cells were subjected to immunocytochemical staining. The cells were found to be positive for the MSC-specific surface markers CD105, CD90, and CD73 and were negative for the hematopoietic cell marker CD45 (Figure-2a).

### *In vitro* differentiation study

The cultured cells were induced to differentiating to osteocytes, chondrocytes, and adipocytes in standard *in vitro* differentiating conditions. After 21 days of culture, the cytochemical staining showed positive results in all culture plates. Osteogenic differentiation was confirmed by the mineralized nodules of orange-red color in alizarin red staining of the culture substrate. Proteoglycan accumulation was noticed as blue color after Alcian blue staining as a confirmation of chondrogenic differentiation. The lipid vacuoles in adipogenic differentiated cells were stained in red color as found in Oil red- O staining (Figure-2b).

## Discussion

UC is considered as one of the easily ­accessible sources of MSCs. Explant culture and enzymatic digestions are the two known techniques for MSCs isolation from UC tissue. Although explant method for obtaining the cells is time taking, the method is safe to generate clinical-grade MSCs because there is a chance of cell membrane damage in other enzymatic digestion methods. Explant culture of gelatinous UCs tissue matrix adjacent to the vessels, a commonly used technique for the isolation of MSCs has been reviewed by Hendijani [18] applicable in different species. In the present study, the explant culture of WJ separated from bovine UCs was done to isolate the cells. We noticed the migration of small spindle-shaped fibroblast-like cells from the edge of the explant within 7 days of culture in MSCs medium that subsequently became confluent within 10 days. Although the timeline varies in different species, appearance of cells from the edges of explant is commonly observed in WJ explant culture [1,14].

In subsequent passages, the isolated cells continually remained adhere to the plastic surface of culture plates and maintained spindle-shaped fibroblastic morphology which is the foremost characteristic feature of MSCs. It has been found that UCs derived MSCs display plastic-adherent fibroblast-like morphology when cultured under identical conditions and also have the capacity to maintain their morphological features even after prolonged culturing at higher passages numbers [19]. The MSCs do have the plastic adherent property and the ability to maintain their fibroblastic morphology is the standard set by the International Society for Cellular Therapy (ISCT) [20].

In a growing population of cells like MSCs, initially for a short interval when the number of cells does not increase much is known as the lag phase. Thereafter, the cell population grows rapidly in an exponential phase followed by the stationary phase where the number of cells no longer increases [21]. In our growth kinetics study, a sharp exponential phase was noticed in both P2 and P4 cells after 24 h of culture lasting up to day 4. The cells of P2 entered into the declining phase immediately after reaching maximum confluence whereas a stationary phase was noticed at the same time in P4 cells. This observation might be due to higher cell density in P2 as compared to P4 on the 4^th^ day of culture. Declining or stationary phase after the 4^th^ day of culture might be due to the confluence of cells in the culture substrates. Hyperdensity of cell population in culture plate might have antagonized cell proliferation due to the natural progression of mitotic division resulting in contact inhibition. Irrespective of origin similar contact inhibition of stem cells at *in vitro* culture condition was established after they reach maximum confluence [4,19,22,23]. We calculated the proliferation and expansion rate of these cells in terms of DT (DT: 1.1767±0.03 in P2 and 1.220±0.01 in P4) which were almost similar to the bMSCs isolated from fetal bone marrow and UC blood [13,24]. Similar to our findings of increased DT value as the passage number progressed was also evident earlier in case of bovine fetal bone marrow-derived MSCs [13].

Substantial effort has been given to the identification of specific surface markers for the selection of MSCs in a cell population. To characterize MSCs, the ISCT has proposed CD105 (Endoglin), CD73 (ecto-5’-nucleotidase), and CD90 (Thy-1) as positive surface markers of human MSCs and lacks the expression of hematopoietic stem cell markers such as CD45, CD34, CD14, and CD11b [20]. Expression of these markers in the MSCs isolated from different sources of other unconventional animal models has been extensively reviewed by Calloni *et al*. [25]. The positive expressions of CD73, CD90, and CD105; and negative expression of CD45 by immunophenotyping here in these isolated bovine UCs derived cells confirmed to meet a criterion of MSCs. Similar pattern in surface marker expression was noticed in the MSCs isolated from different sources of bovine adult tissue [14,16,24].

MSCs are classified as multipotent cells that are capable to differentiate into bone, cartilage, and fat tissues in the presence of appropriate biochemical cue. Such a differentiation capacity also comes under the basic criterion in defining multipotent MSCs [20]. In this present study after incubating the cells under appropriate inducing medium, differentiation was confirmed by the cytochemical staining. Similar *in vitro* differentiation of MSCs followed by its confirmation with cytochemical staining in different species including bovine has been reviewed by Calloni *et al*. [25]. With the evidence of cytochemical staining, we confirmed trilineage differentiation potentiality of bovine UCs derived cells which is considered a criterion of defining MSCs.

## Conclusion

MSCs isolated from diverse sources have been considered as one of the best choices of stem cells for cell-based tissue regeneration. This work was designed to standardize the protocol of isolation of bMSCs minimizing the ethical concern by a non-invasive way from the offcut of UC tissue left over after calving. Cells were isolated by the explant culture of WJ and were maintained *in vitro* with MSC culture medium. These cells exhibited plastic adherent property and maintained spindle-shaped morphology during *in vitro* culture. In immunocytochemical staining, cells were positive for the MSC-specific surface markers CD105, CD90, and CD73; and were negative for the hematopoietic cell marker CD45. The cells were able to differentiate *in vitro* toward osteogenic, chondrogenic, and adipogenic lineages. Taken together, the cells isolated from UC tissue were confirmed to meet the criteria of multipotent MSCs. Therefore, the offcut of bovine UC collected after calving may be considered as one of the easily accessible sources for the isolation of MSCs with the viewpoint of its wide spread availability for the prospective regenerative therapy in cattle.

## Authors’ Contributions

PD: Principal investigator for the execution of entire experiment. TM: Assisted first author in immunocytochemistry work. CM: Sample collection and maintenance of cell line. KK: Assisted first author in cytochemical staining. JM: Quality control of cell line. BCD: Health monitoring of animals during surgical interventions. SB: Data analysis and manuscript preparation. KD: Experiment designing and mentor of the project. All authors have read and approved the final manuscript.
